# CD80 Expressed by CD8^+^ T Cells Contributes to PD-L1-Induced Apoptosis of Activated CD8^+^ T Cells

**DOI:** 10.1155/2017/7659462

**Published:** 2017-10-18

**Authors:** Meagan R. Rollins, Rachel M. Gibbons Johnson

**Affiliations:** ^1^Biology Discipline, University of Minnesota, Morris, MN, USA; ^2^Department of Immunology, Mayo Clinic, Rochester, MN, USA

## Abstract

Tumor cells are capable of limiting antitumor CD8^+^ T cell responses through their cell surface expression of PD-L1. In addition to PD-1 expressed by CD8^+^ T cells, PD-L1 also binds to CD80 expressed by CD8^+^ T cells. The influence of the PD-L1/CD80 interaction on CD8^+^ T cell function has not been fully characterized, so we sought to investigate the impact of the PD-L1/CD80 interaction on PD-L1-induced apoptosis of activated CD8^+^ T cells. We found that CD8^+^ T cells that lacked CD80 expression got activated to the same extent as wild-type CD8^+^ T cells, but when cultured with anti-CD3 and PD-L1/Fc protein, activated CD8^+^ T cells that lacked CD80 expression survived better than activated wild-type CD8^+^ T cells. These findings indicate that PD-L1 induces apoptosis in activated CD8^+^ T cells in part by signaling through CD80. Thus, in the design and implementation of checkpoint blockade therapies that target PD-L1, it is essential that both binding partners for PD-L1, PD-1, and CD80 are considered.

## 1. Introduction

Cell surface expression of the checkpoint protein programmed death ligand 1 (PD-L1, also named B7-H1 and CD274) is a potent mechanism of immune evasion employed by a wide variety of tumor types and is the target of several checkpoint blockade immunotherapies for cancer [1]. PD-L1 limits an antitumor immune response by signaling through its receptors, PD-1 and CD80 (also named B7-1), expressed on the surface of activated CD8^+^ T cells. The influence of the PD-L1/PD-1 interaction on CD8^+^ T cell function has been extensively characterized and is known to limit CD8^+^ T cell responses by inhibiting TCR signaling, thus restricting CD8^+^ T cell survival, proliferation, and cytokine production [2, 3]. The PD-L1/PD-1 interaction is the target of the checkpoint blockade therapies pembrolizumab and nivolumab. Both of these drugs are humanized antibodies that bind to PD-1 and prevent PD-L1 from binding to PD-1, thus eliminating the negative signaling delivered to CD8^+^ T cells by PD-L1 [4, 5]. To date, pembrolizumab is approved for use in metastatic melanoma, both squamous and nonsquamous non-small-cell lung cancer (NSCLC), head and neck squamous cell carcinoma, and Hodgkin's lymphoma. Nivolumab is approved for the treatment of metastatic melanoma, both squamous and nonsquamous NSCLC, and renal cell carcinoma. In clinical trials for both drugs, significant portions of enrolled patients exhibited durable responses or complete tumor elimination [6–14]. There are additional checkpoint blockade therapies, durvalumab, atezolimuab, and avelumab, which bind to PD-L1, blocking the interaction of PD-L1 with both PD-1 and CD80. Currently, durvalumab is approved for the treatment of urothelial carcinoma, atezolimuab is approved for the treatment of NSCLC and urothelial carcinoma, and avelumab is approved for the treatment of Merkel cell carcinoma [15–18]. As durvalumab, atezolimuab, avelumab, and other drugs that target the PD-L1/CD80 interaction in addition to the PD-L1/PD-1 interaction are being designed and implemented, it is necessary to gain a better understanding of how the PD-L1/CD80 interaction is involved in limiting antitumor CD8^+^ T cell responses.

The interaction between PD-L1 and CD80 was first characterized in 2007 [19] but has not been extensively studied since then. The interaction occurs in both mice and humans and has an affinity that is threefold weaker than that of the PD-L1/PD-1 interaction and threefold stronger than that of the CD28/CD80 interaction [19, 20]. When mouse CD4^+^ T cells were cultured with plate-bound PD-L1 and anti-CD3, proliferation and production of proinflammatory cytokines were inhibited, even if the CD4^+^ T cells lacked expression of PD-1 [19]. These results were the first indication that the PD-L1/CD80 interaction functions to limit T cell responses. In related studies, when the PD-L1/CD80 interaction was blocked by an antibody and the PD-L1/PD-1 interaction was left intact, CD8^+^ T cells exhibited an extended period of expansion and decreased induction of anergy in an *in vivo* peptide immunization model [21]. In a cardiac allograft model in mice, specifically blocking the PD-L1/CD80 interaction accelerated graft rejection and led to an increased production of proinflammatory cytokines [22]. Similarly, using the nonobese diabetic mouse model, the blockade of the PD-L1/CD80 interaction accelerated diabetes in older mice [23]. All together, these findings demonstrate that CD80 expressed by T cells can deliver “reverse signaling” into the T cell upon interaction with PD-L1 that is anti-inflammatory and protolerogeneic. Accordingly, tumor cells are likely capable of inhibiting antitumor CD8^+^ T cell responses by signaling through both PD-1 and CD80.

In this study, we specifically investigated the role of PD-L1/CD80 signaling in limiting the survival of activated CD8^+^ T cells. During an immune response, activated CD8^+^ T cells go through a period of expansion; then, after antigen clearance, there is a contraction phase during which a majority of the activated CD8^+^ T cells die by apoptosis. The contraction phase is largely mediated by the mitochondrial pathway of apoptosis [24–26], and we previously demonstrated that PD-L1 signaling is involved in the induction of apoptosis of activated CD8^+^ T cells during the contraction phase. We found that when either the PD-L1/PD-1 interaction or the PD-L1/CD80 interaction was blocked, activated CD8^+^ T cells expressed decreased levels of the proapoptotic protein Bim [27], indicating a novel role for PD-L1/CD80 signaling in limiting the survival of activated CD8^+^ T cells. In this study, CD80-deficient mice were used to demonstrate that the PD-L1/CD80 interaction contributes to the induction of PD-L1-induced apoptosis in activated CD8^+^ T cells. This new information is important to consider in the design and implementation of checkpoint blockade therapies that target PD-L1, as therapies that targetly block the PD-L1 interaction with both PD-1 and CD80 may be more effective than those that only block the PD-L1/PD-1 interaction.

## 2. Materials and Methods

### 2.1. Mice

C57BL/6J wild-type (WT) and CD80-knockout (KO) mice (B6.129S4-Cd80tm1Shr/J) were purchased from Jackson Laboratories. Homozygous CD80-KO mice were bred from heterozygous CD80-KO mice. Mice were used at 6–12 weeks of age. Studies were conducted in accordance with the National Institutes of Health guidelines for the proper use of animals in research and with local Institutional Animal Care and Use Committee approval.

### 2.2. *In Vitro* CD8^+^ T Cell Activation and Culturing with Fusion Proteins

The spleen and lymph nodes of WT and CD80-KO mice were harvested at 6–12 weeks of age. The cells were activated with concanavalin A (ConA, 5 *μ*g/mL, L7647, Sigma-Aldrich) for 48 hours. Following activation, CD8^+^ T cells were purified from the whole cell population (EasySep CD8^+^ T cell negative selection kit, Stem Cell Technologies) and were incubated with plate-bound PD-L1/Fc or recombinant human IgG1/Fc (control/Fc) fusion proteins (R&D Systems) for 48 hours in the presence of anti-CD3 (clone 2C11, BD Biosciences) in ConA-conditioned media (RPMI 1640 medium with L-glutamine and 25 mM HEPES (Lonza) with 10% FBS (Gibco), 1 U/mL penicillin (Gibco), and 1 *μ*g/mL streptomycin (Gibco)). Live cells were counted by Trypan blue (Millipore) exclusion using a hemocytometer.

### 2.3. Western Blotting

Cells were lysed on ice with lysis buffer containing 20 mM Tris, 100 mM NaCl, 1 mM EDTA, 0.5% Triton X-100, and protease inhibitors (Millipore). 0.5 × 10^6^ cells were lysed for each condition and run on SDS-PAGE gels, transferred to nitrocellulose (Bio-Rad), and blotted using standard procedures. Rat anti-mouse Bim mAb (3C5) was purchased from Enzo Life Sciences. Goat anti-rat HRP was purchased from BioLegend. Rabbit anti-mouse actin mAb (D18C11) was purchased from Cell Signaling. Goat anti-rabbit HRP was purchased from Bio-Rad.

### 2.4. Flow Cytometry Analysis

Samples were run on a BD Accuri™ C6 Flow Cytometer and analyzed by BD Accuri C6 Software. For analysis, gates were drawn from live CD8^+^ cells. Fluorochrome-conjugated antibodies against CD8, CD86, and PD-1 were purchased from BioLegend or eBiosciences.

### 2.5. Statistical Analysis

A two-sided paired Student's *t*-test was used to assess statistical differences in experimental groups. A *p* value < 0.05 was considered statistically significant.

## 3. Results

### 3.1. Characterization of CD8^+^ T Cells Activated in the Absence of PD-L1/CD80 Signaling

**Figure 1:**
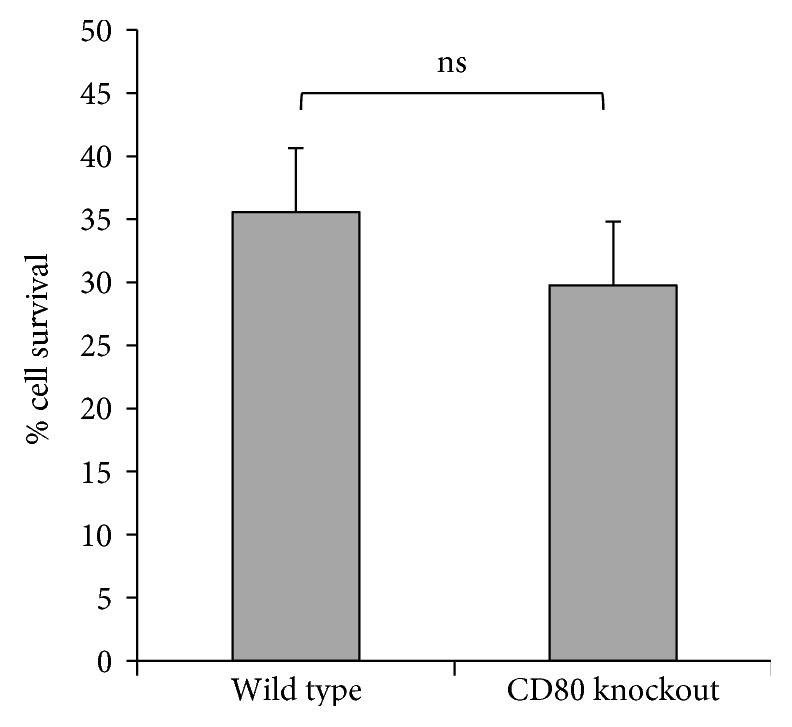
CD80-KO and WT CD8^+^ T cells survive at equal levels after ConA activation. WT and CD80-KO cells were activated with ConA, then harvested for analysis. Live cells were counted by Trypan blue exclusion (*n* = 3, ±SD, *p* = 0.24, ns: not significant).

**Figure 2:**
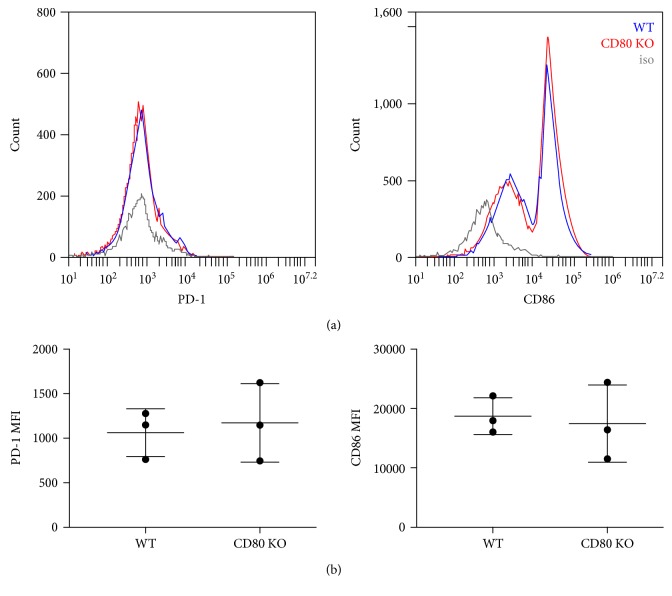
CD80-KO and WT CD8^+^ T cells express equivalent levels of cell surface markers of activation after ConA activation. WT and CD80-KO cells were activated with ConA, then harvested for analysis. Cells were then analyzed by flow cytometry. (a) Histograms are of live CD8^+^ cells and representative of 3 separate experiments. (b) Mean fluorescent intensity (MFI) for PD-1 and CD86 (*n* = 3, ±SD, not significant).

In order to investigate the influence of PD-L1/CD80 signaling on activated CD8^+^ T cell survival using CD80-KO CD8^+^ T cells, we first needed to determine whether or not CD80-KO and WT CD8^+^ T cells were activated equivalently. We used an *in vitro* culture system in which splenocytes were harvested from naïve WT and CD80-KO mice and activated for 48 hours with ConA. Cells were then harvested for analysis. CD80-KO and WT CD8^+^ T cells survived at equivalent levels after ConA activation as shown in Figure 1. We also assessed the expression of cell surface markers of activation, including CD86 and PD-1, and found that CD80-KO and WT CD8^+^ T cells expressed equivalent levels of these markers after *in vitro* activation (Figure 2). Since PD-1 expression was equivalent between CD80-KO and WT CD8^+^ T cells, it appears that the defect in CD80 expression in the CD80-KO CD8^+^ T cells does not affect the expression of PD-1 by these cells. Based on these findings, we concluded that CD8^+^ T cells get activated in our *in vitro* culture system equivalently in the absence of PD-L1/CD80 signaling.

### 3.2. Activated CD8^+^ T Cells Survive Better in the Absence of PD-L1/CD80 Signaling

**Figure 3:**
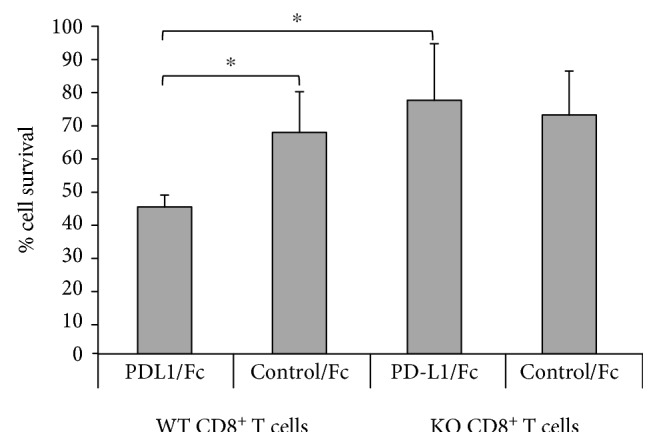
CD80-KO CD8^+^ T cells survive better than WT CD8^+^ T cells when cultured with PD-L1/Fc. ConA-activated WT and CD80-KO CD8+ cells were cultured with anti-CD3 and either recombinant mouse PD-L1/Fc or control/Fc for 48 hours, then harvested for analysis. Live cells were counted by Trypan blue exclusion (*n* = 3, ±SD, ^∗^*p* ≤ 0.05).

We next went on to investigate the influence of PD-L1/CD80 signaling on the survival of activated CD8^+^ T cells. We used the same *in vitro* activation with ConA as above; then, after harvesting the activated cells, we isolated the activated CD8^+^ T cells and cultured them for an additional 48 hours on plates coated with anti-CD3 and either PD-L1/Fc or control/Fc protein. Cells were then harvested for analysis. Activated CD80-KO and WT CD8^+^ T cells cultured with anti-CD3 and control/Fc protein were recovered at equal levels after the culture period, but more activated CD80-KO CD8^+^ T cells cultured with anti-CD3 and PD-L1/Fc protein were recovered than activated WT CD8^+^ T cells cultured with anti-CD3 and PD-L1/Fc (Figure 3). These data indicate that PD-L1/CD80 signaling limited the survival of activated CD8^+^ T cells.

### 3.3. Bim Expression Is Decreased in Activated CD8^+^ T Cells in the Absence of PD-L1/CD80 Signaling

**Figure 4:**
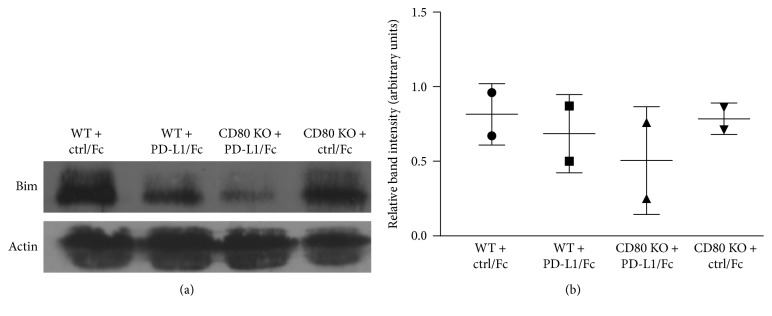
Decreased Bim protein levels in activated CD80-KO CD8^+^ T cells cultured with PD-L1 as compared to WT cells. (a) ConA-activated WT and CD80-KO CD8^+^ cells were cultured with anti-CD3 (clone 2C11) and either recombinant mouse PD-L1/Fc or control/Fc (ctrl/Fc) for 48 hours, then harvested for analysis. Cells were lysed and analyzed by Western blotting for Bim and actin protein levels. Representative of two separate experiments. (b) Bim signals were quantified using ImageJ and normalized to actin signals (*n* = 2, ±SD, not significant).

We went on to investigate the mechanism by which PD-L1/CD80 signaling limited the survival of activated CD8^+^ T cells. CD80-KO and WT CD8^+^ T cells were activated and cultured with anti-CD3 and PD-L1/Fc protein as described above; then, the expression levels of the proapoptotic protein Bim were analyzed by Western blotting. As shown in Figure 4(a), activated CD80-KO CD8^+^ T cells cultured with anti-CD3 and PD-L1/Fc protein expressed decreased levels of Bim as compared to WT cells. This finding was supported by two separate experiments. The Bim signals from the Western blots were quantified using ImageJ and normalized to actin signals as shown in Figure 4(). This finding demonstrates that PD-L1/CD80 signaling contributes to the induction of apoptosis in activated CD8^+^ T cells by inducing increased expression of Bim.

## 4. Discussion

Activated CD8^+^ T cells are potent killer cells but are themselves very sensitive to being killed by apoptosis. The PD-L1/PD-1 signaling pathway is well known to induce apoptosis of activated CD8^+^ T cells, but the contribution of the PD-L1/CD80 signaling pathway to apoptosis of activated CD8^+^ T cells has not been extensively investigated. In this study, we demonstrate that PD-L1/CD80 signaling contributes to the induction of apoptosis of activated CD8^+^ T cells by inducing increased expression of Bim. We used an *in vitro* ConA activation system for our studies and first confirmed that CD80-KO and WT CD8^+^ T cells get activated to the same extent (Figures 1 and 2). We found that there are no intrinsic differences between CD80-KO and WT CD8^+^ T cells upon activation. We then went on to culture the ConA-activated CD8^+^ T cells with PD-L1 and found that the CD80-KO CD8^+^ T cells survived better than the WT CD8^+^ T cells (Figure 3). The increased survival of the CD8^+^ T cells that lacked PD-L1/CD80 signaling was due, at least in part, to decreased levels of Bim expression (Figure 4).

The goal of checkpoint blockade therapies that target PD-L1 expressed by tumor cells is to reactivate an antitumor CD8^+^ T cell response; thus, it is crucial that we fully understand the mechanisms by which PD-L1 signaling limits antitumor CD8^+^ T cell responses. Based on our findings reported here, if a checkpoint blockade therapy only inhibits the PD-L1/PD-1 signaling pathway and leaves the PD-L1/CD80 signaling pathway intact, then PD-L1 expressed by tumor cells will still be able to induce apoptosis of tumor-infiltrating CD8^+^ T cells by signaling through CD80. It has also been reported that PD-L1 limits CD8^+^ T cell responses in part by inhibiting glycolysis downstream of PD-1 signaling [28, 29]. It was reported that PD-L1/PD-1 blockade led to a metabolic reprogramming in activated CD8^+^ T cells that resulted in increased rates of glycolysis. This metabolic switch induced by the PD-L1/PD-1 blockade in CD8^+^ T cells was due to increased Akt activation in the absence of PD-1 signaling. Bim expression levels are also regulated by Akt signaling in CD8^+^ T cells [30], so it is possible that PD-L1/CD80 signaling, in addition to influencing Bim expression levels, may also influence the metabolism of CD8^+^ T cells. Continued studies into the influence of the PD-L1/CD80 pathway on CD8^+^ T cell functions are necessary.

## Abbreviations

ConA: Concanavalin A
NSCLC: Non-small-cell lung cancer
PD-L1: Programmed death ligand 1
KO: Knockout
WT: Wild type.
